# Differences in Supragingival Microbiome in Patients with and without Full-Crown Prostheses

**DOI:** 10.3390/dj10080152

**Published:** 2022-08-15

**Authors:** Manli Guo, Zhidong Zhang, Jiyuan Lu, Di Wang, Yimin Yan, Shen Zhang, Xin Yu, Songhua Su, Lu Yuan, Zhige Li, Baoping Zhang

**Affiliations:** 1 Key Lab of Oral Diseases of Gansu Province, Northwest Minzu University, Lanzhou 730000, China; 2 School of Stomatology, Lanzhou University, Lanzhou 730000, China; 3 Hospital of Stomatology Lanzhou University, Lanzhou 730000, China

**Keywords:** oral microorganism, community structure, full-crown prosthesis, 16S rRNA high-throughput sequencing

## Abstract

**Objectives:** To characterize the microflora profile of supragingival biofilm in patients with and without full-crown prostheses. **Methods:** Plaque samples of full-crown prostheses and teeth in patients with porcelain-fused-to-metal crowns, all-ceramic crowns, and no prostheses were collected (three patients per group), using 16S rRNA high-throughput sequencing technology to conduct DNA sequencing on the samples and using Qiime, R, and PICRUSt2 software to perform bioinformatics analyses and functional analyses on sequencing data. **Results:** In total, 110,209 valid sequences were obtained in the experiment, corresponding to 11 phyla and 120 genera. The predominant species shared by the three groups were phyla *Actinobacteria*, *Bacteroidetes*, *Firmicutes*, *Fusobacteria*, and *Proteobacteria* and genera *Rothia*, *Porphyromonas*, *Prevotella*, *Streptococcus*, *Veillonella*, *Leptotrichia*, *Neisseria*, *Citrobacter*, and *Pseudomonas*. The species-difference analysis showed that genus *Hameophilus* significantly increased after the patient wore the dental prosthesis. Compared with the no-prosthesis samples, the functional analysis showed that cell motility increased in the samples from full-crown prostheses, while replication and repair, and translation decreased. **Conclusions:** This study reveals the changes in the oral microbial community of patients with full-crown prostheses, which could provide insights regarding the safety of materials for long-term use in the oral cavity.

## 1. Introduction

As an important detecting site of the human microbiome project, the oral cavity is the second largest microbial gathering area after the intestinal tract, where as many as 1000 species of bacteria can be found [1,2]. With the development of the ecological plaque theory in oral microbial research [3,4,5], it has been found that the oral microbial community is closely related to the occurrence of oral diseases and the evolution of humans [6,7]. When the oral microbial community changes from a balanced coexistence of multiple bacteria to a dominance of few pathogenic bacteria (such as genera *Veillonella* and *Prevotella* [8]), it may affect the immune response and metabolism of the host and cause serval diseases, such as diabetes, bacteremia, endocarditis, cancer, autoimmune diseases, premature birth, etc. [9,10]. Obviously, the abundance, species, and interactions of microorganisms are crucial for oral microbiology research. Studies have found that an individual’s oral health, body mass index, age, and other factors cause changes in the quantity of bacteria in or species of the oral microbial community [8]. Studies have confirmed that caries, periodontitis, oral neoplasms, and the failure of clinical treatment are related to the plaque biofilm formed by the accumulation of oral microorganisms [11]. Hybrid ceramic and zirconia-reinforced lithium disilicate materials have excellent fracture resistance compared with composite resins and other materials and have become commonly used oral restorative materials [12]. After wearing dental prosthesis in the oral cavity, dental-prosthesis materials may cause changes in the oral microbial community’s structure, and different microorganisms that use saliva as a medium can gather on the surface of the dental prosthesis, leading to the vigorous development of plaque biofilm and affecting the growth and development of bacterial and life metabolism activities, which are closely related to oral health and disease conditions [7,13,14,15]. Therefore, paying attention to the influence of dental-prosthesis materials on the micro-environment of the oral cavity is a major focus of current research in the field of microbiology that helps to monitor the safety of materials for long-term use in the oral cavity.

The study of microbial diversity has traditionally mainly relied on isolation, culture, and identification technology. Although cultured strains can be obtained, they provide little insight into the ecological interaction among microorganisms and are not sufficient to study the composition of the microbial community. Moreover, the success rate of culturing bacteria in the laboratory is extremely low [16]. High-throughput fingerprinting technology has overcome the difficulties of microbial culture; it describes the dynamics and trends of the microbial community through the differences in PCR-amplification-fragment sizes of the 16S rRNA gene with electrophoresis equipment. However, these techniques can only detect the most abundant few species in the community and may lead to PCR deviation [17]. The latest DNA-sequence-based microarray technology can illuminate a larger dynamic range of microbial communities than fingerprinting technology and can perform the comparation among complex samples, but the limitations of the use of probes hinders the comprehensive investigation of microbial diversity [18]. In recent years, 16S rRNA, known as “bacterial fossils”, has dominated the bacterial analyses based on sequencing due to its moderate gene size (about 1542 bp in length) and low mutation rate. 16S-amplicon-sequencing technology has become the common method of molecular biological identification [19] and is widely used in the etiological studies of dental caries and periodontal disease.

The aim of the present study was to characterize the microflora profile of supragingival biofilm in patients with or without full-crown prostheses. We intended to collect plaque samples of full-crown prostheses and teeth in patients with porcelain-fused-to-metal crowns, all-ceramic crowns, and no prostheses and then use 16S rRNA high-throughput sequencing to reveal the oral microbial community structure and diversity of different full-crown prostheses, which can provide experimental evidence for the long-term safe use of dental prostheses and its impact on the oral microbial community.

## 2. Materials and Methods

### 2.1. Patients

**Figure 1:**
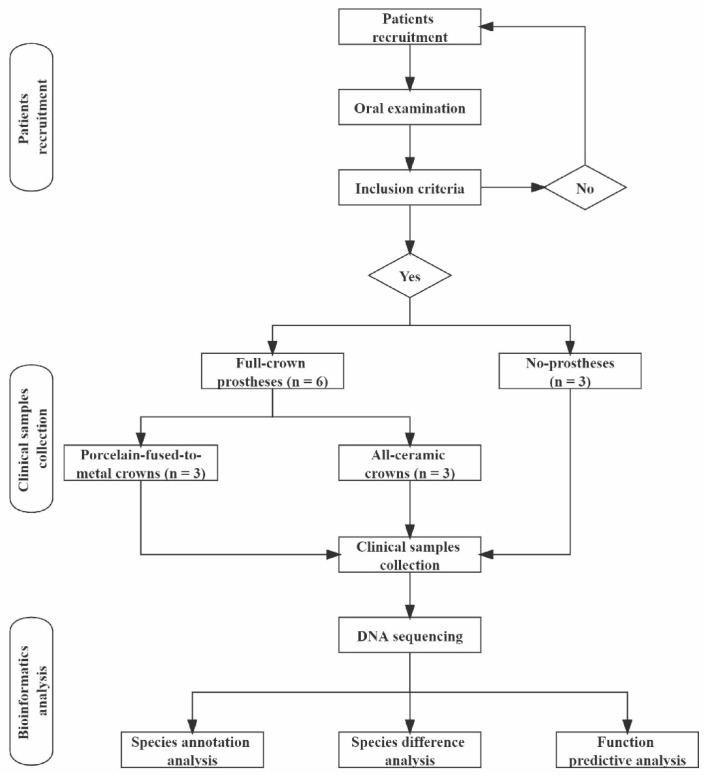
Experimental flow chart.

We selected patients with first molars needing full-crown restoration treatment in Department of Prosthodontics of Hospital of Stomatology Lanzhou University from June to July 2021 and divided them into no prostheses (HG), porcelain-fused-to-metal crowns (which are dental prostheses of gold–porcelain-composite structure fused to a metal-base crown obtained by low-melting porcelain under vacuum conditions) (CG), and all-ceramic crowns (which are dental prostheses made of all-ceramic materials that cover the entire surface of the dental crown) (AG) groups. A total of 9 patients were included, with 3 patients in each group. The experimental process is shown in Figure 1. This study was approved by the ethics committee of School of Stomatology Lanzhou University (LZUKQ-2020-028) and performed in accordance with the principles stated in the Declaration of Helsinki (amended by the 64th WMA General Assembly, Fortaleza, Brazil, October 2013). All patients were fully informed about our research project before providing samples and signed informed consent forms.

Inclusion criteria: 30–60 years old; first molars needing full-crown restoration treatment; number of remaining teeth in the mouth ≥ 20; oral health status (caries status, DMFT ≤ 4; periodontal status, detected gingival-bleeding teeth ≤ 8; detected periodontal-pocket teeth ≤ 2; no deep periodontal pockets; no loss of attachment); no smoking, drinking, or other bad habits; no antibiotics in the past three months.

Exclusion criteria: patients with systemic disease; receiving antibiotics or antifungal drugs; using steroids (systemic or inhaled), immunosuppressants, or investigational drugs; participating in another clinical study 30 days before recruitment.

### 2.2. Clinical-Sample Collection and DNA Isolation

Oral examinations were performed on the patients to assess the number of remaining teeth in the mouth, the caries status (DMFT), and the periodontal status (gingival bleeding, periodontal pockets, loss of attachment). The eligible patients were selected according to our inclusion and exclusion criteria. Then, we collected plaque samples of full crowns 3–6 months after restoration treatment in the groups porcelain-fused-to-metal crowns and all-ceramic crowns. Moreover, plaque samples from the teeth adjacent to the defected teeth were collected in the no-prosthesis group. Plaque samples were collected by wiping the surface of the prostheses and teeth with a cotton swab for 10 s. All collected samples were stored with PBS buffer (pH = 7.4) in an enzyme-free EP tube and frozen in a −80 °C refrigerator.

DNA isolation was performed using a bacterial genomic DNA extraction kit (Solarbio), strictly following the instructions. DNA purity (OD260/280) and DNA concentration were detected with Nanodrop (Thermo), and DNA integrity was detected with an electrophoresis apparatus.

### 2.3. High-Throughput Sequencing

After the DNA samples passed the inspection, PCR amplification was performed using forward primer 338F (5′-ACTCCTACGGGAGGCAGCAG-3′) and reverse primer 806R (5′-GGACTACHVGGGTWTCTAAT-3′); then, the PCR product was subjected to the establishing of the gene library and sequencing on the PacBio platform.

### 2.4. Statistical and Bioinformatics Analyses

Demographic data and clinical data were statistically analyzed with SPSS 20.0 software. All measurement data were expressed as mean ± standard deviation, and the comparison of the means among groups was performed with a one-way ANOVA. For the data not conforming to the normal distribution, a non-parametric test was adopted. The level of statistical significance was set at *p* < 0.05.

Qiime software was used to perform the OTU clustering of valid DNA sequences with a similarity threshold of 97% [20]. According to the NCBI 16S ribosomal RNA (Bacteria and Archaea) database [21], the RDP Classifier Bayesian algorithm was used for the taxonomic analysis of the OTUs with a credibility of 80% [22]. The dilution curve was used to reflect the rationality of sequencing data. The alpha-diversity index and the rank–abundance curve were used to reflect species richness and their uniformity. The similarities and differences among microbial communities were analyzed with a PCoA analysis.

The VennDiagram package in R software was used to draw a Venn diagram for counting the number of common or unique OTU among different samples (groups). The ggplot2 package in R software was used to draw the histogram of the relative abundance of species and then select the species ranked top 30 to perform the similarity clustering of the relative abundance, which was shown with a heat map drawn with the pheatmap package in R software [23]. LEfSe software was used to perform a linear discriminant analysis (LDA) on the taxa with significant differences in abundance, and a Metastats analysis was used to detect species differences at the phylum and genus levels, so as to find the species with significant differences in sample classification [24]. PICRUSt2 (Phylogenetic Investigation of Communities by Reconstruction of Unobserved States) is software that predicts the functional abundance of a sample based on the abundance of the marker-gene sequences in the sample; this was used to predict the functions of the microbial community in each group [25].

## 3. Results

### 3.1. General Outline

A total of nine patients were included in this study, including five females and four males. The average ages of the three groups were 39.00 ± 8.72, 43.67 ± 9.29, and 42.33 ± 11.93, respectively, and there were no significant differences among the groups (*p* = 0.847). In addition, the patients in each group had good oral health statuses and had no bad habits, such as smoking and drinking, as shown in Table 1.

A total of 116,198 original sequences were obtained in the experiment. After primer removal and length filtering (1300~1600 bp), 110,209 valid sequences were obtained, and the details of each sample’s sequences are shown in Table S1. After the OTU clustering of these valid sequences, 339 original OTUs were obtained; then, 336 OTUs after leveling were used for subsequent analyses. As shown in the dilution curve (Figure S1a), when the sequencing depth reached 9000, the curves tended to be flat, indicating that the number of the samples’ sequencing data was reasonable and could reflect the vast majority of flora information. The results in Table 2 show that the species richness of the three groups was as follows: porcelain crowns, no prostheses, and all-ceramic crowns. However, the difference was not statistically significant (*p* = 0.277), and there were no significant differences in the uniformity of species classification among the three groups, as shown in Figure S1b and Table S2.

### 3.2. Species Annotation and Diversity in Samples from Porcelain-Fused-to-Metal Crowns, All-Ceramic Crowns, and No Prostheses

**Figure 2:**
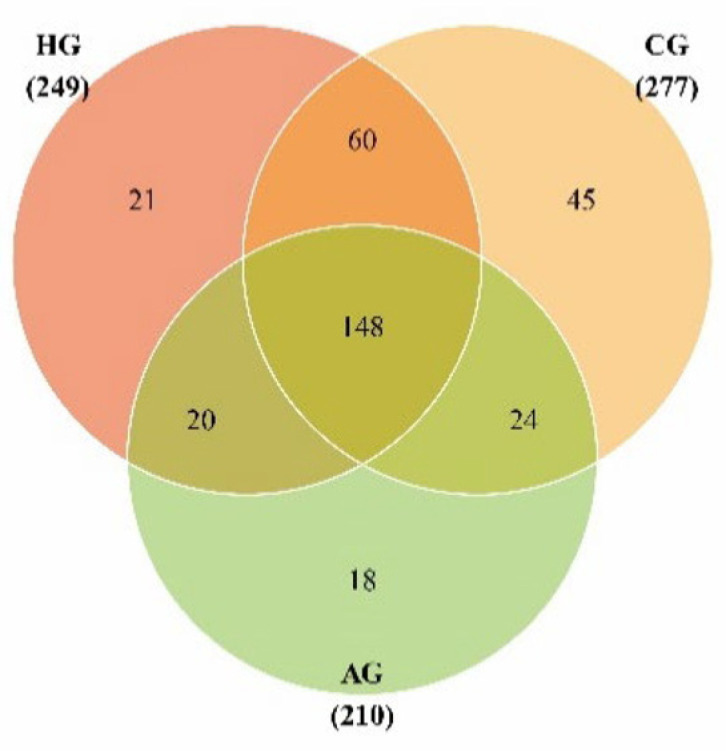
Venn diagram of the 3 groups.

**Figure 3:**
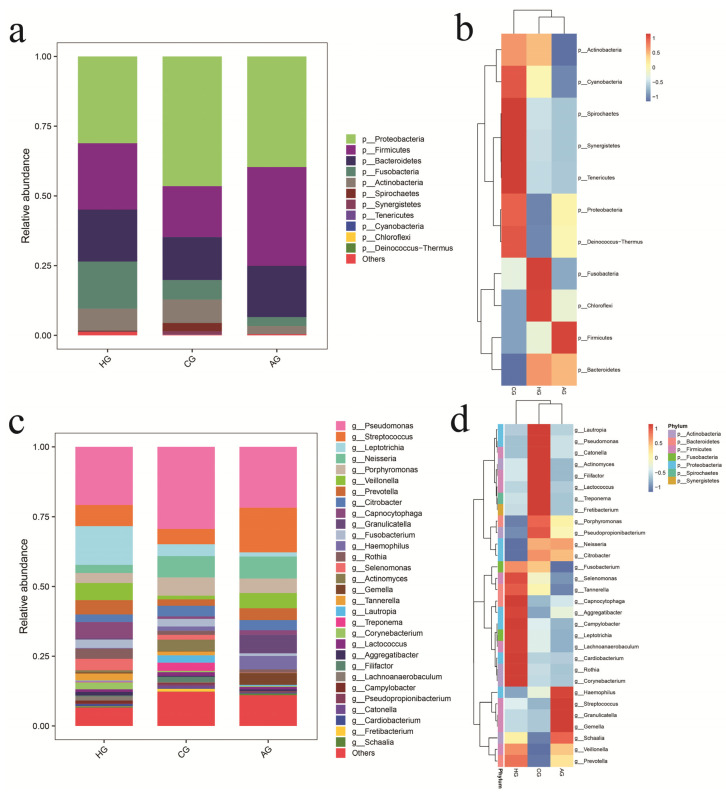
Histogram of species abundance at phylum (**a**) and genus (**c**) levels and heat map of species abundance at phylum (**b**) and genus (**d**) levels.

Figure 2 shows the OTU distributions for the three groups, and there were 44.05% OTUs in common among them. After species annotation, OTUs could be classified into 1 kingdom, 11 phyla, 23 classes, 44 orders, 74 families, 120 genera, and 188 species, as shown in Table S3. The dominant species (˃1%) were shared by three groups and were: phyla *Proteobacteria*, *Firmicutes*, *Bacteroidetes*, *Fusobacteria*, and *Actinobacteria* and genera *Pseudomonas*, *Streptococcus*, *Leptotrichia*, *Neisseria*, *Porphyromonas*, *Veillonella*, *Prevotella*, *Citrobacter*, and *Rothia*. The proportion of phylum *Proteobacteria* in the samples from full-crown prostheses was higher than that in the no-prosthesis samples, but the proportion of phylum *Fusobacteria* in the no-prosthesis samples was higher than that in the samples from full-crown prostheses, as shown in Figure 3a,c.

To access the similarities and differences in the community composition of the three groups at the phylum and genus taxonomic levels, we performed a heatmap analysis (Figure 3b,d). At the phylum level, the communities in the all-ceramic-crown and no-prosthesis samples had a close evolutionary relationship, but the communities in the all-ceramic-crown and porcelain-crown samples had a close evolutionary relationship at the genus level.

**Figure 4:**
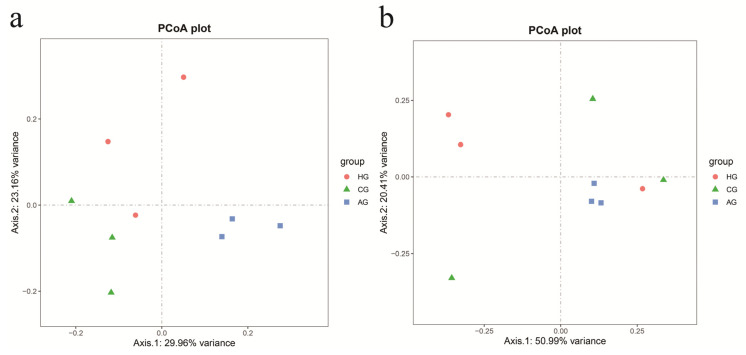
Beta-diversity analysis of the samples performed using the Principal Co-ordinates Analysis based on the unweighted Unifrac distance (**a**) and the weighted Unifrac distance (**b**). The more similar the community composition of the samples was, the closer their distance in the PCoA diagram is.

Figure 4 shows the beta-diversity analysis of the nine samples. The PCoA analysis based on the unweighted Unifrac distance showed that the three groups were clustered respectively and were at a certain distance with respect to each other. The distances among the samples within the groups were also slightly scattered, indicating that the changes in the oral flora were greatly affected by individual differences. The PCoA analysis based on the weighted Unifrac distance showed that there was some overlap among the groups, indicating that there were similar flora among the three groups. The results of the Anosim multivariate analysis of variance showed that there were differences in species diversity among the three groups (*p* = 0.009), but there were no significant differences between the two groups (*p* > 0.05), as shown in Figure S2.

### 3.3. Species-Difference Analysis of Samples from Porcelain-Fused-to-Metal Crowns, All-Ceramic Crowns, and No Prostheses

**Figure 5:**
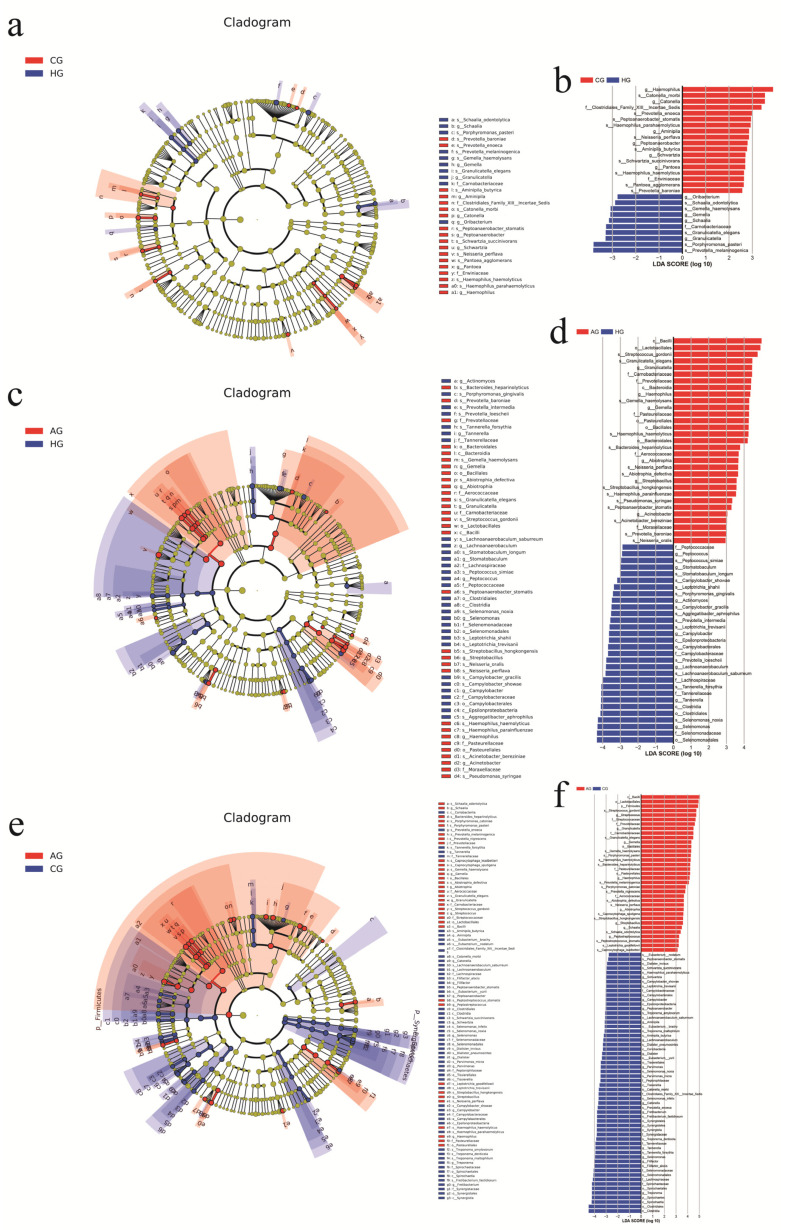
Species-difference analysis of different groups. (**a**,**c**,**e**) Colors represent different groups. The red nodes in the branches represent microbial groups that play an important role in the red groups, and the green nodes represent microbial groups that play an important role in the green groups. Yellow nodes represent microbial taxa that did not play a significant role in either group. (**b**,**d**,**f**) The LDA value distribution diagram of the different species, the LDA value distribution histogram shows the species whose LDA Score is greater than the set value (the default setting is 2), that is, the Biomarker with statistical difference between groups. The length of the histogram represents the effect size of the different species.

In the porcelain-fused-to-metal-crown and no-prosthesis groups, there were 11 phyla in total, and there were no statistically significant differences between two groups (*p* > 0.05). There were 116 genera, and 8 genera were significantly different between the two groups. Genera *Pantoea*, *Peptoanaerobacter*, *Aminipila*, *Haemophilus*, and *Parvimonas* in porcelain-fused-to-metal-crown samples were found to be present at significantly higher levels than those in the no-prosthesis samples (*p* < 0.05), but genera *Gemella*, *Granulicatella*, and *Schaalia* in the no-prosthesis samples were found to be present at significantly higher levels (*p* < 0.05), as shown in Figure 5a,b and Table S4.

In the all-ceramic-crown and no-prosthesis groups, there were 11 phyla, and there were no statistically significant differences between two groups at the phylum level (*p* > 0.05). There were 102 genera in the two groups, and the difference between two groups was statistically significant in 9 genera; genera *Gemella*, *Haemophilus*, *Granulicatella*, *Acinetobacter*, *Abiotrophia*, and *Peptostreptococcus* in the all-ceramic-crown samples were found to be present at significantly higher levels than those in the no-prosthesis samples (*p* < 0.05), but genera *Peptococcus*, *Stomatobaculum*, and *Actinomyces* were found to be present at significantly higher levels in the no-prosthesis samples (*p* < 0.05), as shown in Figure 5c,d and Table S5.

In the all-ceramic-crown and porcelain-fused-to-metal-crown groups, there were 11 phyla, and phylum *Firmicutes* in the all-ceramic-crown samples was found to be present at a significantly higher level than that in the porcelain-fused-to-metal-crown samples (*p* < 0.05). There were 119 genera, and 14 genera showed statistically significant differences between the two groups; genera *Gemella*, *Granulicatella*, *Schaalia, Streptococcus*, *Peptostreptococcus*, *Haemophilus*, *Abiotrophia*, and *Veillonella* in the all-ceramic-crown samples were found to be present at significantly higher levels than those in the porcelain-fused-to-metal-crown samples (*p* < 0.05), but genera *Peptoanaerobacter*, *Parvimonas*, *Aminipila*, *Campylobacter*, *Lachnoanaerobaculum*, and *Selenomonas* in the porcelain-fused-to-metal-crown samples were found to be present at significantly higher levels (*p* < 0.05), as shown in Figure 5e,f and Table S6.

### 3.4. Function-Predictive Analysis of Microbial Community

**Figure 6:**
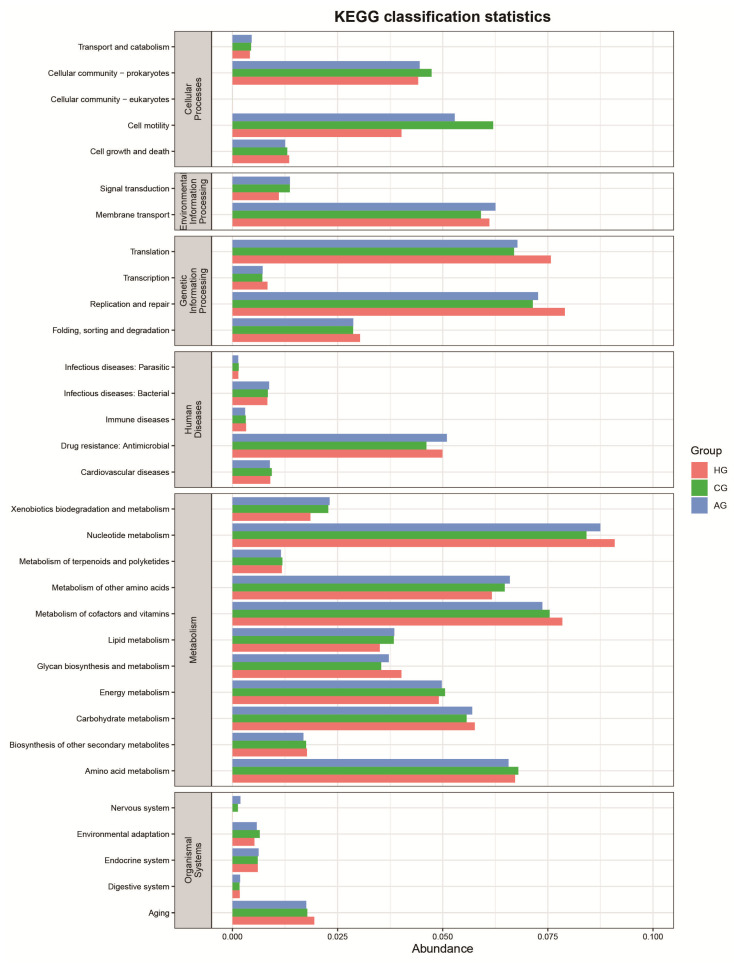
Function-predictive analysis of different groups.

The core of the KEGG database is the biological metabolic pathway analysis database; biological pathways are mainly concentrated in six kinds: cellular processes, environmental-information processing, genetic-information processing, human diseases, metabolism, and organismal systems. Figure 6 shows that cell motility (cellular process) was lower in the no-prosthesis group than in the full-crown-prosthesis group, but replication and repair, and translation (genetic-information processing) in the no-prosthesis group were higher.

## 4. Discussion

Full-crown restoration is the main treatment for tooth or dentition defects [26,27]. Porcelain crowns are low cost and easy to clean, and all-ceramic crowns have good biocompatibility and aesthetic appearance; both are still commonly used in dental prostheses. A large body of research has indicated that the stimulation of the restoration margin and subgingival plaque leads to the content of gingival crevicular fluid, and inflammatory factors such as IL-6 can be significantly increased after wearing porcelain-fused-to-metal crowns for a period of time [28,29]. As a multifunctional cytokine, IL-6 can promote the aggregation of inflammatory cells and the release of inflammatory mediators. It can also inhibit the growth of periodontal ligament cells, collagen cells, and stromal cells; weaken the repair ability of the periodontal ligament and the attachment ability of fibroblasts; and eventually form deep periodontal pockets [30,31]. Therefore, periodontal tissue is inevitably damaged to varying degrees after full-crown restoration, and Deinzer et al. pointed out that the species and numbers of bacteria could reflect the destruction of periodontal tissues [32]. Moreover, different microorganisms in the oral cavity also use saliva as a medium to accumulate on the surface of a full-crown restoration to form supragingival-plaque biofilm. There are few studies on the changes in the oral bacteria flora after full-crown restoration, and most of them are static analyses of single bacterial species [33]. To elucidate the influence of dental prostheses on the diversity of the oral microbiome, this study completed the species-difference analyses and function-predictive analyses of dental plaque collected in patients with porcelain crowns, all-ceramic crowns, and no prostheses using high-throughput sequencing technology.

The beta-diversity analysis showed that the distribution of the samples from porcelain crowns and no prostheses was relatively discrete, while the distribution of the three samples from all-ceramic crowns was more concentrated, indicating that the oral microbial diversity of the patients with porcelain crowns and no prostheses was higher than that of patients with all-ceramic crowns. However, the alpha-diversity analysis indicated that there were no statistically significant differences in microbial diversity among the three groups. It indicates that the oral microbiome is complex and diverse whether a dental prosthesis is worn or not, which further supports the theory of oral microecological plaque [3]. In addition, the species annotated from the sequencing data were concentrated in phyla *Actinobacteria*, *Bacteroidetes*, *Firmicutes, Fusobacteria*, and *Proteobacteria*, which were the same as the core microbes in healthy individuals found by Egija Zaura et al. [34]; this is also consistent with the result of the study by Digvijay Verma et al. [10] that the 16S rDNA map of healthy oral cavities divided the parasitic bacteria into phyla *Firmicutes*, *Actinobacteria*, *Proteobacteria*, *Fusobacteria*, *Bacteroidetes,* and *Spirochaetes*, accounting for 96% of the total oral bacteria. It showed that there were no noticeable changes in the oral flora of full-crown prostheses at the phylum level.

Genera *Rothia*, *Porphyromonas*, *Prevotella*, *Streptococcus*, *Veillonella*, *Leptotrichia*, *Neisseria*, *Citrobacter*, *Haemophilus*, and *Pseudomonas* were the common genera in full-crown prostheses. Yu et al. [35] found that genus *Gemella* was the dominant genus after complete denture restoration, and we found that genera *Gemella*, *Granulicatella*, and *Haemophilus* were more frequently found in the all-ceramic-crown samples than in the no-prosthesis samples. Moreover, Egija Zaura et al. [34] first observed the diversity and uniqueness of an individual’s oral microbial community and proposed the concept of core microbial community in healthy oral cavities. They found that *Firmicutes* (*Streptococcus*, *Veillonellaceae*, *Granulicatella*), *Proteobacteria* (*Neisseria*, *Haemophilus*), *Actinobacteria* (*Corynebacterium*, *Rothia*, *Actinomyces*), *Bacteroidetes* (*Prevotella*, *Capnocytophaga*, *Porphyromonas*), and *Fusobacteria* (*Fusobacterium*) were dominant in healthy individuals. In our study, genera *Veillonella*, *Leptotrichia*, *Citrobacter*, and *Pseudomonas* in the oral cavity of patients with full-crown prostheses became the dominant genera in comparison with healthy people. The transformation of these dominant genera may break the microecological balance of the healthy oral cavity and ultimately lead to the occurrence of oral diseases. The species-difference analysis showed that genus *Haemophilus* in the oral cavity of patients with full-crown prostheses increased significantly compared with patients with no prostheses. Genus *Haemophilus* mainly resides in the airway and oral cavity of humans or animals, causing primary purulent infections and severe secondary infections, and studies have shown that it is closely related to airway inflammation, such as allergic airway inflammation [36]. In our study, genus *Haemophilus* was the dominant genus in the oral cavity of patients with full-crown prostheses, which may cause the secondary inflammation of periodontal tissues around the prostheses. In addition, studies have found that the main oral bacterial flora of patients with denture stomatitis were genera *Prevotella*, *Streptococcus*, and *Atopobium* [37,38]. Our study found that genera *Prevotella* and *Streptococcus* were the dominant genera in the full-crown-prosthesis and no-prosthesis samples, which shows that genera *Prevotella* and *Streptococcus* were shared by healthy people and patients with denture stomatitis after denture restoration [35].

Of course, the community structure and diversity of microorganisms also reflect the functional characteristics of microbial communities. Moreover, some studies have attempted to link specific gene-function-expression profiles of oral bacteria with the establishment and maturation of oral biofilm [39,40]. There are two important mechanisms in the initial stage of biofilm formation: cell movement and cell adhesion. Studies have found that motility plays an important role in the formation of biofilm structures [41]. Microorganisms being close to the prosthesis or tooth surface is the first step in the formation of biofilm. The functional-prediction analysis showed that the cell motility of the full-crown prostheses was higher, which indicated that the surface of the prostheses was more likely to form biofilm. The genetic-information-processing function was higher in the no-prosthesis samples, indicating that protein-synthesis and -degradation activities were more active in the no-prosthesis group.

Our study had several limitations, including the small sample of nine patients and the lack of in-depth verification research of the subgingival-microbial-diversity distribution and mechanisms. This study revealed the growth and decline of oral normal bacteria and pathogenic bacteria after full-crown restoration and described the changes in the oral microbial community in patients with full-crown prostheses. We found that the wearing of a dental prosthesis did not change the diversity of the oral microbial community; it only broke the balance of the oral microecology and changed the composition of the original microbial community. Moreover, genus *Hameophilus* was the dominant genus that significantly increased after full-crown restoration. Although, following recent biomaterial research, new biomaterials such as Phosphorene or Borophene can be widely used in prosthetic coatings to improve the antibacterial properties of prosthetics due to their excellent antibacterial properties [42,43], the results of the present study also suggest that the oral microbial community should be considered in the development of oral materials for long-term use in clinical practice.

## Figures and Tables

**Table 1:** Demographic and clinical data of HG, CG, and AG.

Characteristics	HG (n = 3)	CG (n = 3)	AG (n = 3)	*p*-Value
Gender (M/F)	1/2	2/1	1/2	
Age (Mean ± SD (years))	39.00 ± 8.72	43.67 ± 9.29	42.33 ± 11.93	0.847
Number of missing teeth (Mean ± SD)	24.33 ± 2.08	23.67 ± 1.53	23.33 ± 2.08	0.815
DMFT (Mean ± SD)	1.00 ± 1.00	1.33 ± 1.15	1.33 ± 1.53	0.932
Number of detected gingival-bleeding teeth (Mean ± SD)	3.00 ± 3.00	4.33 ± 2.08	4.00 ± 2.00	0.787
Number of detected periodontal-pocket teeth (Mean ± SD)	0.33 ± 0.58	0.67 ± 0.58	0.67 ± 1.15	0.850
Number of detected attachment-loss teeth	0	0	0	

Note: HG, no prostheses; CG, porcelain-fused-to-metal crowns; AG, all-ceramic crowns.

**Table 2:** Characteristics of sequencing data.

Group	Valid CCS Sequences	OTUs	chao1	Shannon
HG	13,996 ± 2896	155 ± 41	186.5 ± 51.8	4.6 ± 0.8
CG	10,718 ± 891	187 ± 17	234.8 ± 53.2	4.4 ± 1.2
AG	10,718 ± 891	138 ± 23	170.9 ± 26.6	4.4 ± 0.3

Note: HG, no prostheses; CG, porcelain-fused-to-metal crowns; AG, all-ceramic crowns.

## Data Availability

The datasets generated and/or analyzed during the current study are available from the corresponding author upon reasonable request. The data are not publicly available due to privacy reasons with respect to the subjects in the study.

## Supplementary Materials

The following supporting information can be downloaded at: https://www.mdpi.com/article/10.3390/dj10080152/s1, Table S1: Detail sequencing data of nine samples; Table S2: Species diversity index of nine samples; Table S3: Species annotation of nine samples; Table S4: Species difference analysis between porcelain-fused-to-metal-crowns and non-prostheses; Table S5: Species difference analysis between all-ceramic crowns and and non-prostheses; Table S6: Species difference analysis between porcelain-fused-to-metal-crowns and all-ceramic crowns; Figure S1: Sample dilution curve (a) and rank abundance curve (b) of samples; Figure S2: Anoism similarity analysis among different groups.

## Abbreviations

HP1, HP2, HP3:	The three samples from patients with no prostheses.
CPH, CPR, CPF:	The three samples from patients with porcelain crowns.
APH, APR, APF:	The three samples from patients with all-ceramic crowns.
