# Supplementary material for: Differences in Supragingival Microbiome in Patients with and without Full-Crown Prostheses
Source: Dent J (Basel). 2022 Aug 15;10(8):152. doi: 10.3390/dj10080152 (PMC9406617; doi:10.3390/dj10080152)
Supplement: Supplementary file 1 [file dentistry-10-00152-s001.zip › dentistry-1784159-supplementary.pdf]

Table S1 Detail sequencing data of nine samples.

group	sample	original CCS sequences	CCS sequences after primer removal	valid CCS sequences	average length of valid CCS sequences (bp)
	CPH	11203	10521	10510	1461
CG	CPR	10905	9971	9950	1460
	CPF	12167	11705	11695	1453
	APH	11004	10527	10517	1461
AG	APR	12949	12132	12107	1461
	APF	14134	13439	13433	1464
	HP1	17972	17212	17200	1454
HG	HP2	11999	11569	11565	1453
	HP3	13865	13254	13222	1459

CCS：Circular Consensus Sequences.

Table S2 Species diversity index of nine samples.

group	sample	Chao1	shannon	observed-species	ace	simpson	PD-whole-tree
	CPH	291.5	4.54	197.0	270.14	0.92	27.25
CG	CPR	226.7	3.14	197.0	230.78	0.75	29.29
	CPF	186.1	5.53	168.0	183.81	0.95	25.06
	APH	195.2	4.78	163.0	199.93	0.93	24.86
AG	APR	175.0	4.39	134.0	185.31	0.92	21.10
	APF	142.5	4.16	117.0	155.04	0.92	18.47
	HP1	182.0	5.24	151.0	181.69	0.96	22.94
HG	HP2	137.1	4.79	116.0	139.09	0.93	17.18
	HP3	240.5	3.68	198.0	246.15	0.79	26.67

Table S3 Species annotation of nine samples.

group	sample	kingdom	phylum	class	order	family	genus	species
	CPH	1	10	19	32	52	79	129

CG	CPR	1	10	21	36	58	83	124
	CPF	1	9	18	30	47	67	101
	APH	1	10	18	30	47	68	102
AG	APR	1	7	18	27	48	65	91
	APF	1	8	14	24	41	53	77
	HP1	1	9	18	27	39	58	90
HG	HP2	1	6	13	24	35	48	77
	HP3	1	10	18	31	51	78	130

Table S4 Species difference analysis between porcelain crowns and no-prostheses.

taxonomy	species	mean		P value
		CG	HG	
phylum	Firmicutes			
genus	Peptoanaerobacter	0.002	0.0004	0.014
genus	Gemella	0.001	0.004	0.017
genus	Granulicatella	0.0005	0.004	0.027
genus	Aminipila	0.0004	0	0.034
genus	Parvimonas	0.006	0.002	0.045
phylum	Proteobacteria			
genus	Pantoea	0.0003	0.0001	0.013
genus	Haemophilus	0.016	0.002	0.035
phylum	Actinobacteria			
genus	Schaalia	0.0005	0.004	0.046

Table S5 Species difference analysis between all-ceramic crowns and and no-prostheses.

taxonomy	species	mean		P value
		AG	HG	
phylum	Firmicutes			
genus	Gemella	0.042	0.004	＜0.001
genus	Peptococcus	0	0.0005	0.001
genus	Granulicatella	0.066	0.004	0.015

genus	Stomatobaculum	0	0.0004	0.016
genus	Abiotrophia	0.009	0.0004	0.042
genus	Peptostreptococcus	0.004	0.001	0.048
phylum	Proteobacteria			
genus	Haemophilus	0.048	0.002	0.008
genus	Acinetobacter	0.0002	0	0.018
phylum	Actinobacteria			
genus	Actinomyces	0.001	0.008	0.021

Table S6 Species difference analysis between porcelain crowns and all-ceramic crowns.

taxonomy	species	mean		P value
		CG	AG	
phylum	Firmicutes	0.183	0.354	＜0.001
genus	Gemella	0.001	0.042	＜0.001
genus	Peptoanaerobacter	0.002	0.0004	0.002
genus	Granulicatella	0.0005	0.066	0.008
genus	Parvimonas	0.006	0.00003	0.009
genus	Streptococcus	0.055	0.160	0.017
genus	Peptostreptococcus	0.0007	0.004	0.018
genus	Aminipila	0.0004	0	0.027
genus	Abiotrophia	0	0.009	0.034
genus	Lachnoanaerobaculum	0.003	0.00003	0.037
genus	Veillonella	0.013	0.054	0.038
genus	Selenomonas	0.017	0.001	0.040
phylum	Actinobacteria			
genus	Schaalia	0.0005	0.006	0.014
phylum	Proteobacteria			
genus	Haemophilus	0.016	0.048	0.028
genus	Campylobacter	0.002	0.0004	0.036

Figure S1 Sample dilution curve (a) and rank abundance curve (b) of samples. (a) When the curve

tends to be flat, it indicates that the amount of sequencing data is reasonable. On the contrary, it indicates that the continued sequencing may also generate more new OTUs. (b) The richness of the classification in the horizontal direction is reflected by the width of the curve, and the higher the richness of the classification, the larger the span of the curve on the horizontal axis. The smoothness of the curve in the vertical direction reflects the uniformity of the classification in the sample, and the smoother the curve, the more even the species distribution.

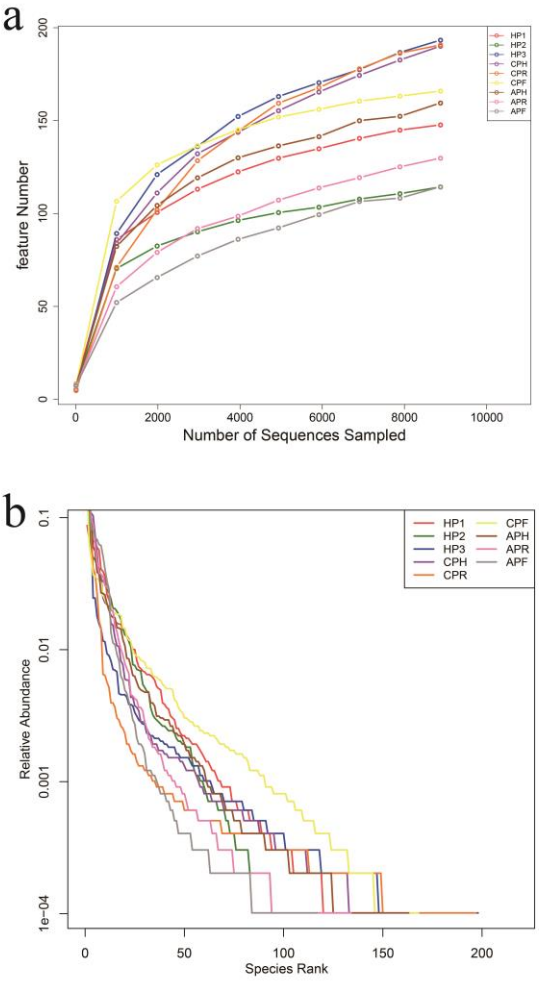

Figure S2 Anoism similarity analysis among different groups.

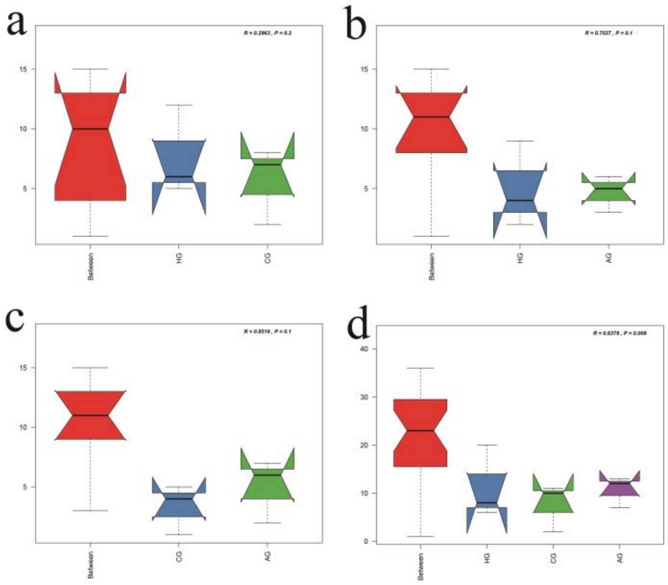
a
R = 0.2963 , P = 0.2
15
10
5
Between
HG
CG
b
R = 0.7037 , P = 0.1
15
10
5
Between
HG
AG
c
R = 0.8519 , P = 0.1
15
10
5
Between
CG
AG
d
R = 0.6379 , P = 0.009
40
30
20
10
0
Between
HG
CG
AG
